# Pain medication use in patients with X-linked hypophosphatemia treated with burosumab: a retrospective real-world study

**DOI:** 10.1093/jbmrpl/ziag047

**Published:** 2026-03-24

**Authors:** Carolyn M Macica, Zhiyi Li, Ben Johnson, Ahmed Noman, Catherine Park, Gina N Woods

**Affiliations:** University of Connecticut School of Medicine and Connecticut Children’s Research Institute, Department of Research Operations and Development, Hartford, CT 06106, United States; Kyowa Kirin Inc., HEOR, Princeton, NJ 08540, United States; Kyowa Kirin International, HEOR, Marlow SL7 1HZ, United Kingdom; Komodo Health Inc., New York, NY 10010, United States; Komodo Health Inc., New York, NY 10010, United States; UC San Diego Health, School of Medicine, CA 92093–0602, United States

**Keywords:** X-linked hypophosphatemia, pain, pain medication, opioids, US claims data, burosumab

## Abstract

X-linked hypophosphatemia (XLH) is a rare, progressive phosphate-wasting disorder that leads to significant skeletal morbidity and pain. The current study used real-world administrative claims data to assess the demographic and clinical characteristics of patients with XLH related to claims for prescription pain medication (PPM) during the 12 mo before starting burosumab treatment (baseline) and explores changes in PPM claims after starting burosumab treatment. Claims for XLH-related musculoskeletal and deformity-related conditions, obesity, and osteoarthritis were common among the adult cohort (*n* = 387). Claims for PPM (vs no claims) during baseline were significantly (*p* < .05) associated with: age ≥ 50 yr (odds ratio [OR] 2.54), Medicaid coverage (2.25), myalgia (2.54), scoliosis (3.19), obesity (2.07), dental complications (2.53), and physical therapy (2.94). Approximately half of the adult cohort (54%) made PPM claims during baseline, and 38% for opioids, with little change after starting burosumab. Claims for arthralgia, hip/leg deformities and rickets were common in pediatric patients (*n* = 524). Claims for PPM were significantly associated with race (OR 0.13), chronic pulmonary disease (4.34), renal disease (4.88), arthralgia (2.20), cholecalciferol claims (1.93), and physical therapy (3.48). About one-fifth of pediatric patients (25%) had claims for PPM prescriptions during baseline, with little change after 12 mo, but showed a modest decrease during the second year of follow-up; 12%-17% of pediatric patients had claims for opioids during baseline and follow-up. The number of days covered by opioid prescriptions was lower in pediatric patients than in adults. Multiple factors are associated with PPM use in patients with XLH. Understanding these factors may inform patient-centered pain management strategies. A longer study is needed to determine how burosumab treatment affects pain and the use of PPM in patients with XLH.

## Graphical Abstract

**Graphical Abstract:**
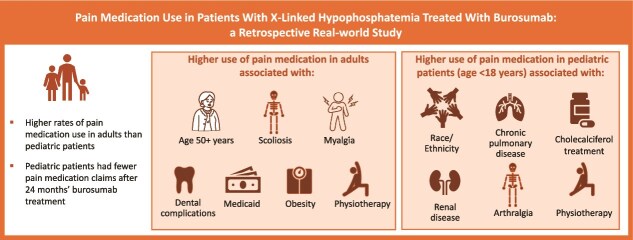

## Introduction

X-linked hypophosphatemia (XLH) is a rare, genetic, lifelong, progressive disorder caused by excess FGF23 as a result of mutations in the *PHEX* (phosphate regulating endopeptidase X-linked) gene. This leads to renal phosphate wasting and decreased levels of 1,25(OH)2D3.^1^ Chronic hypophosphatemia during childhood causes dysregulated bone metabolism, which compromises skeletal development, leading to gait abnormalities (which may require surgical correction), delayed and disproportionate growth, and short stature.^2–4^ Children with XLH often experience skeletal and muscle pain.^2^^,^^5^^,^^6^ Skeletal deformities that develop during childhood become irreversible when bone growth stops, and persistent hypophosphatemia in adulthood may lead to the accumulation of further skeletal morbidities, including osteomalacia, osteoarthritis, enthesopathies, spinal stenosis, and pseudofractures.^7–11^ Adults with XLH frequently report pain, stiffness, and impaired physical function.^6^^,^^8^ The symptoms of XLH also have a marked impact on activities of daily living,^8^^,^^10^^,^^12–14^ work productivity,^15^ and health-related quality of life.^8^^,^^12^ XLH may not be diagnosed until adulthood in some patients, with the potential for uncontrolled disease progression without appropriate management.

Pain is a significant issue for patients with XLH. In a survey of 232 adults and 90 children with XLH, 97% of adults and 80% of children reported experiencing bone or joint pain in the previous year and muscle pain was reported by 63% of adults and 60% of children.^6^ Pain varies widely in severity, type, frequency, and location, both within and between patients.^6^^,^^8^^,^^13^ Age at onset of pain varies, but severity typically worsens over time.^8^ One study found bone pain to be more frequent in children, whereas joint pain was more prominent in adults.^10^ Patients with XLH use a variety of strategies to manage their pain, including prescription and over-the-counter pain medication^6^^,^^8^^,^^16^ and nonpharmacological strategies.^8^^,^^16^ Pain has been reported to compromise physical functioning, daily activities, psychological well-being, and social life.^8^^,^^10^^,^^16^

Burosumab is a neutralizing Ab to FGF23, targeting the underlying cause of XLH. It was approved by the Food and Drug Administration in April 2018, and is indicated in the United States for the treatment of XLH in adults and pediatric patients (aged ≥6 mo).^17^ In clinical trials, burosumab increased serum phosphate levels in both pediatric and adult patients, significantly reduced the severity of rickets in children, and improved patient-reported stiffness, pain, and physical functioning, and fracture/pseudofracture healing in adults.^18–22^ The treatment benefit is maintained with long-term treatment (up to 184 wk in adults and 160 wk in children).^23–25^ However, a recent real-world study reported that while treatment with burosumab was associated with improvements in patient-reported pain scores, there was no change in the use of pain medication.^26^

The high frequency of pain in patients with XLH, and its broad negative impact, may indicate a need for better patient-centered pain management strategies. To achieve this, a greater understanding of clinical characteristics and the use of pain medication in these patients is needed. While XLH studies show pain is a major challenge for patients, few studies report on the patients’ use of pain medication. The current study uses real-world administrative claims data to assess the demographic and clinical characteristics of pupatients with XLH that are related to claims for prescription pain medications (PPM).

The objectives of the current analysis were to describe the demographic and clinical characteristics of adult and pediatric patients with XLH before starting burosumab treatment, and to identify demographic and clinical characteristics that may be associated with PPM claims (and specifically opioids). A supplementary analysis explored changes in the use of PPM (and opioids [± non-opioids] separately) and pain-related services among adults and pediatric patients with XLH during the first 2 yr of starting burosumab treatment.

## Materials and methods

### Study design

This was a retrospective study using the Komodo Research Dataset (KRD). The KRD captures routinely collected health services utilization records and expenditures for more than 330 million unique de-identified individuals in the United States from disparate sources (clearing houses, payers, and providers), providing patient-level observations of medical encounters and outpatient pharmacy dispensing via linkage across health and pharmacy insurance plans. While the KRD includes both open and closed claims, the current analysis used only closed claims; these are claims that have been submitted to an insurer, adjudicated, and paid. This type of claim provides a high level of detail and accuracy but is limited to a patient’s time within a specific insurer.^11^ Claims are based on International Classification of Diseases, 10th Edition (ICD-10) codes.

The study period was from April 17, 2017 (1 yr before burosumab was approved in the United States [April 17, 2018]), to April 30, 2024 (end of study; EoS). The index date was the first claim for burosumab.

**Figure 1:**
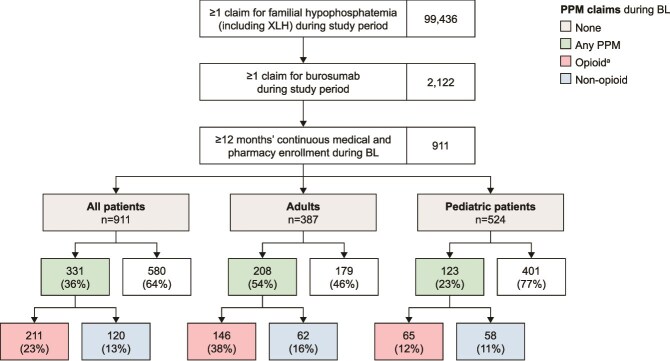
Patient flow. ^a^Patients with claims for opioids could also have claims for non-opioids. Patients with XLH were identified as those having at least 1 claim for familial hypophosphatemia (International Classification of Disease, 10^th^ edition, code E8331) AND at least 1 claim for burosumab (Healthcare Common Procedure Coding System code J0584; National Drug Codes 69794010201, 69794020301, 69794030401, 42747010 201, 42747020301, 42747030401). Study period was from April 17, 2017 (1 yr before FDA approval of burosumab) to April 30, 2024. Baseline (BL) refers to the 12 mo before the first dose of burosumab (index date). FDA, Food and Drug Administration; PPM, prescription pain medication; XLH, X-linked hypophosphatemia.

As there is no specific ICD-10 Clinical Modification code for XLH, patients with XLH were identified as those having at least 1 claim for familial hypophosphatemia AND at least 1 claim for burosumab (see Figure 1) between April 17, 2018 and EoS. Familial hypophosphatemia may include other conditions besides XLH but burosumab is only indicated for the treatment of XLH (among the familial hypophosphatemias); this combination of claims should, therefore, identify patients with XLH who were prescribed burosumab.

### Outcomes

Patient demographics (age, sex, race/ethnicity, index year, and payer channel) were collected on the index date (first claim for burosumab) and are reported separately for adults (≥18 yr) and pediatric patients (<18 yr).

Clinical characteristics of adult and pediatric patients were collected for the baseline period (the 12 mo preceding the index date) for patients who were continuously enrolled for medical and pharmacy benefits during that period. Clinical characteristics comprised the Charlson Comorbidity Index (CCI) score and its constituent comorbidities,^27^ musculoskeletal manifestations, non-musculoskeletal manifestations (depression, diabetes, and obesity), deformity-related conditions (Chiari malformation, craniosynostosis, delayed growth/walking, short stature, hip/leg-related deformities, and rickets), other disease-related symptoms/conditions (vitamin D deficiency [which has a well-documented association with XLH], dental complications, difficulty walking, muscle weakness, hearing loss, hyperparathyroidism, kidney stones, nephrocalcinosis, osteomalacia, tinnitus, and vertigo), treatments for XLH (excluding burosumab), and the use of pain-related healthcare services: physical therapy (PT), occupational therapy, and alternative medicine (acupuncture and chiropractic services).

Claims for PPM during baseline are reported separately for adults and pediatric patients who had any claims for PPM, claims for opioids ± non-opioids, or claims for non-opioids only. The number of non-overlapping cumulative days covered by prescriptions and the opioid dose (calculated as the morphine-equivalent daily dose) are also reported.

### Statistical analysis

Patient demographics are reported as the number of subjects, mean, and SD for continuous variables, and by the number and percentage of patients for categorical variables. Clinical characteristics are reported as the number and percentage of patients.

Bivariate and multivariate analyses were used to identify associations between demographic and clinical characteristics with PPM claims during baseline for the following groups:

- patients with and without claims for PPM
- patients with claims for opioids (± non-opioids) vs patients with no opioid claims (ie, claims for non-opioids or no PPM claims).

Separate analyses were conducted for the adult and pediatric cohorts.

Bivariate analyses (Wilcoxon rank sum test; Pearson’s Chi-squared test with a simulated *p* value [based on 2000 replicates]) were used to evaluate potential associations between claims for PPM and demographic and clinical characteristics.

Multivariate logistic regression models were used to assess relationships between PPM claims and demographic and clinical characteristics in separate models for adult and pediatric patients. Variables that were significant in the bivariate analysis (*p* < .05) were included in the multivariate analysis. Selection of variables also took into account multicollinearity to prevent inflated coefficient estimates and to aid model convergence, and adequate sample size. For variables that were both continuous and categorical in the bivariate analysis (eg, age), only the categorical variables were included, to avoid multicollinearity. The multivariate logistic regression model simultaneously adjusted for all included demographic and clinical variables, isolating the unique contribution of each factor while controlling for potential confounding effects.

Changes in claims for PPM and pain-related services are reported descriptively for adults and pediatric patients who had data for the baseline and the following 12 mo, and those who had data for the baseline and the following 24 mo.

## Results

### Sample selection

Patient flow is illustrated in Figure 1. Of 99 436 patients with claims for familial hypophosphatemia during the study period, 2122 also had claims for burosumab and were, therefore, determined to have XLH. Of these, 911 patients had the required 12 mo’ continuous medical and pharmacy enrollment during the baseline period, comprising 387 adults and 524 pediatric patients.

### Baseline characteristics of patients

The baseline demographic characteristics of patients treated with burosumab (ie, before treatment started) are presented in Table 1; clinical characteristics are presented in Table 2.

**Table 1:** Baseline (pre-treatment) demographic characteristics of adult (≥18 yr) and pediatric (<18 yr) patients with XLH who had claims for burosumab treatment.

		**Adults (*n* = 387)**	**Pediatric patients (*n* = 524)**
**Age, years**	Mean ± SD	37.8 ± 14.9	8.9 ± 4.7
	Range	18–82	1–17
**Age group, years, *n* (%)**			
** <11**		–	340 (64.9)
** 12–17**		–	184 (35.1)
** 18–29**		138 (35.7)	–
** 30–39**		78 (20.2)	–
** 40–49**		84 (21.7)	–
** ≥50**		87 (22.5)	–
**Sex, *n* (%)**			
** Female**		263 (68.0)	307 (58.6)
** Male**		118 (30.5)	214 (40.8)
** Unknown**		6 (1.6)	3 (0.6)
**Year started burosumab (index year), *n* (%)**			
** 2018^a^**		29 (7.5)	54 (10.3)
** 2019**		111 (28.7)	176 (33.6)
** 2020**		69 (17.8)	77 (14.7)
** 2021**		64 (16.5)	88 (16.8)
** 2022**		61 (15.8)	78 (14.9)
** 2023**		42 (10.9)	45 (8.6)
** 2024^a^**		11 (2.8)	6 (1.1)
**Payer channel, *n* (%)^b^**			
** Commercial**		238 (61.5)	186 (35.5)
** Managed Medicaid/Medicaid**		105 (27.1)	332 (63.4)
** Medicare Advantage/FFS**		41 (10.6)	<3
** Other/unknown**		3 (0.8)	4 (0.8)
**Race and ethnicity, *n* (%)**			
** Asian or Pacific Islander**		8 (2.1)	12 (2.3)
** Black or African American**		34 (8.8)	72 (13.7)
** Hispanic or Latino**		44 (11.4)	82 (15.6)
** White**		206 (53.2)	229 (43.7)
** Other/unknown**		95 (24.5)	129 (24.6)

Abbreviations: FFS, fee for service; HIPAA, Health Insurance Portability and Accountability Act; XLH, X-linked hypophosphatemia.

a 2018 and 2024 were incomplete years.

b Payer channel categories (commercial health insurance; Medicaid [joint federal–state program covering medical costs for low-income individuals of any age] and Medicare Advantage/FFS [federal health insurance for individuals >65 yr of age and younger individuals with disabilities]) were mutually exclusive.

Counts <3 have been masked to comply with HIPAA regulations.

**Table 2:** Baseline (pre-treatment) clinical characteristics of adult (≥18 yr) and pediatric (<18 yr) patients with XLH who had claims for burosumab treatment.

		**Adults (*n* = 387)**	**Pediatric patients (*n* = 524)**
**CCI score**			
** Mean ± SD**		0.5 ± 1.1	0.2 ± 0.7
** Range**		0–8	0–11
**CCI comorbidities, *n* (%)^a^**			
** Chronic pulmonary disease**		55 (14.2)	48 (9.2)
** Diabetes without complications**		21 (5.4)	4 (0.8)
** Renal disease**		26 (6.7)	15 (2.9)
**Musculoskeletal manifestations, *n* (%)**			
** Arthralgia**		292 (75.5)	202 (38.6)
** Enthesopathy**		111 (28.7)	18 (3.4)
** Fracture**		115 (29.7)	62 (11.8)
** Kyphosis**		8 (2.1)	3 (0.6)
** Myalgia**		94 (24.3)	25 (4.8)
** Osteoarthritis**		217 (56.1)	10 (1.9)
** Scoliosis**		39 (10.1)	57 (10.9)
** Spinal stenosis**		82 (21.2)	5 (1.0)
**Non-musculoskeletal manifestations, *n* (%)**			
** Depression**		168 (43.4)	72 (13.7)
** Hypertension**		179 (46.3)	37 (7.1)
** Obesity**		248 (64.1)	172 (32.8)
**Deformity-related conditions, *n* (%)**			
** Chiari malformation**		18 (4.7)	15 (2.9)
** Craniosynostosis**		<3	43 (8.2)
** Delayed growth/delayed walking**		<3	99 (18.9)
** Hip/leg-related deformities^b^**		75 (19.4)	373 (71.2)
** Rickets**		249 (64.3)	434 (82.8)
** Short stature**		30 (7.8)	223 (42.6)
**Other symptoms and conditions of interest, *n* (%)**			
** Vitamin D deficiency**		248 (64.1)	179 (34.2)
** Dental complications**		71 (18.3)	168 (32.1)
** Difficulty walking**		110 (28.4)	134 (25.6)
** Muscle weakness**		74 (19.1)	61 (11.6)
** Hearing loss**		101 (26.1)	75 (14.3)
** Hyperparathyroidism**		99 (25.6)	25 (4.8)
** Kidney stone**		44 (11.4)	19 (3.6)
** Nephrocalcinosis**		42 (10.9)	78 (14.9)
** Osteomalacia**		66 (17.1)	3 (0.6)
** Tinnitus**		48 (12.4)	5 (1.0)
** Vertigo**		15 (3.9)	<3
**Treatments, *n* (%)**			
** Phosphate salts/active vitamin D**	Calcitriol	255 (65.9)	370 (70.6)
	Phosphate supplements	161 (41.6)	217 (41.4)
** Other conventional vitamin D**	Cholecalciferol	55 (14.2)	125 (23.9)
**Pain-related healthcare service utilization**			
** Physical therapy**	Patients with visits, *n* (%)	97 (25.1)	95 (18.1)
	Number of visits, mean ± SD	14.3 ± 13.9	12.2 ± 14.7
** Occupational therapy**	Patients with visits, *n* (%)	19 (4.9)	20 (3.8)
	Number of visits, mean ± SD	3.5 ± 7.1	1.5 ± 1.1
** Alternative medicine^c^**	Patients with visits, *n* (%)	29 (7.5)	6 (1.1)
	Number of visits, mean ± SD	12.0 ± 11.3	6.7 ± 4.4

Abbreviations: CCI = Charlson Comorbidity Index; HIPAA = Health Insurance Portability and Accountability Act; XLH = X-linked hypophosphatemia.

a CCI comorbidities with claims by ≥5% of patients in either age group are reported.

b Genu varum, genu valgum, varus deformities, and coxa vara.

c Acupuncture and chiropractic services.

Clinical characteristics were assessed during baseline (the 12 mo before the first prescription for burosumab [the index date]) for patients with continuous medical and pharmacy benefits during that period.

Counts <3 have been masked to comply with HIPAA regulations.

#### Adults

The adult cohort (*n* = 387) was two-thirds female and just over half were White (Table 1). The mean age was 37.8 yr (range 18-82). Approximately one-third of patients were in the youngest adult age group (18-29 yr), with the remainder evenly distributed across the other age groups. Almost two-thirds (62%) were covered by commercial medical insurance, and 27% by Medicaid.

Adults with XLH showed a substantial burden of musculoskeletal and related clinical conditions at baseline (Table 2). Overall comorbidity was low, with a mean CCI score of 0.5, although a few patients had claims for chronic pulmonary and renal disease. Claims for musculoskeletal manifestations were common, particularly arthralgia and osteoarthritis, and many adults also had claims for fractures, enthesopathy, myalgia, and spinal stenosis. Deformity-related features were also evident in adults, including those affecting the hips and legs. Systemic and pain-relevant conditions were also frequent, including obesity, hypertension, depression, vitamin D deficiency, dental complications, and tinnitus.

Two-thirds of adults had claims for calcitriol during the baseline period, and nearly half for phosphate supplements. One-quarter had claims for PT during the baseline year, typically with multiple visits (the mean was about 1 visit per month), whereas occupational therapy and alternative medicine services were claimed for less often.

Just over half of adults had at least 1 claim for PPM during baseline, more frequently for opioids than non-opioids (Figure 1). Approximately one-third made no claims for PPM during this period.

#### Pediatric patients

The pediatric cohort (*n* = 524) was 59% female, and the majority were White (44%) (Table 1). They had a mean age of 8.9 yr (range 1-17) and almost two-thirds were in the younger age group (≤11 yr). Almost two-thirds (63%) were covered by Medicaid, and 36% by commercial medical insurance.

Pediatric patients with XLH showed a lower overall comorbidity than adults (mean CCI score 0.2), although, as in adults, a few patients had claims for chronic pulmonary and renal disease. The frequency of musculoskeletal conditions at baseline was lower in pediatric patients than in adults (Table 2). The most common conditions were arthralgia, fractures, and scoliosis. However, claims for deformity-related conditions were much higher in the pediatric cohort than in adults, notably rickets, leg/hip-related deformities (genu varum, genu valgum, varus deformities, and coxa vara), and short stature. One-fifth had claims for delayed growth/walking. Rates for systemic and pain-relevant conditions were generally lower in the pediatric cohort than in adults, other than dental complications, for which there were almost twice as many claims. Obesity and vitamin D deficiency were the most common of these conditions in the pediatric cohort, affecting approximately one-third.

Claims for calcitriol and phosphate supplements were similar in the pediatric and adult cohorts, but the pediatric cohort had fewer claims for PT, occupational therapy, or alternative medicine.

Just less than one-quarter of the pediatric cohort had claims for PPM during the baseline period, with similar proportions prescribed opioids (with or without non-opioids) and non-opioid pain medication only (Figure 1).

### Comparison of patients with and without prescription pain medication claims before burosumab treatment

#### Adults

In the bivariate analysis comparing adults with claims for PPM vs no claims, the following demographic variables were significant (Table S1): age and age group on the index date and payer channel. Numerous clinical variables were significant (Table S2): CCI score (and, among the CCI-specific comorbidities, diabetes without complications, mild liver disease, and rheumatic disease), all the musculoskeletal conditions considered except kyphosis, and all 3 non-musculoskeletal conditions (hypertension, obesity, and depression). Among the deformity-related conditions considered, only “rickets” was significant; among other disease-related symptoms/conditions, dental complications, difficulty walking, and muscle weakness were significant. (Claims for “rickets” are not age-restricted and may appear in adult claims [see Table S2]; XLH may be coded as vitamin D-resistant rickets, given the absence of a specific ICD-10 code.) For the use of pain-related healthcare services, claims for PT or occupational therapy visits and the number of PT visits were significant.

**Figure 2:**
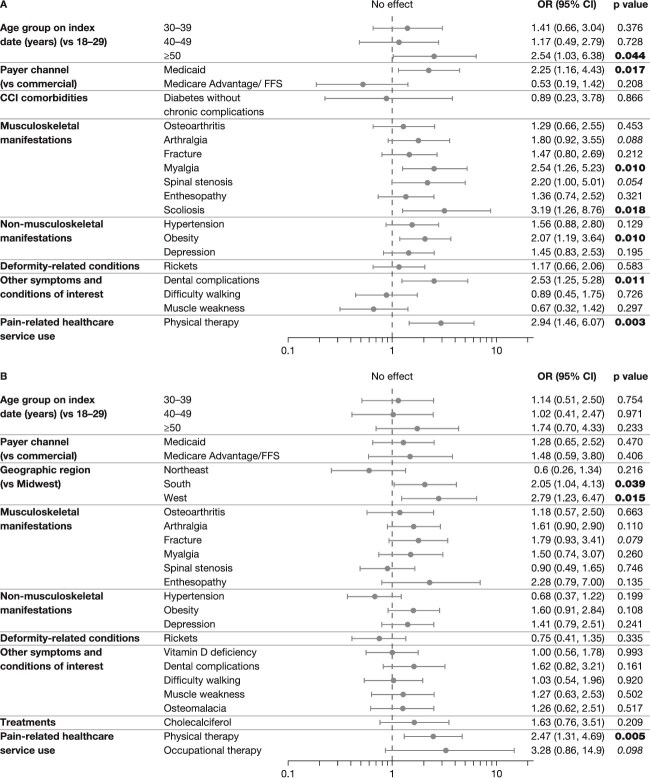
Results of the multivariate analysis comparing adults with XLH before burosumab treatment: (A) claims vs no claims for PPM; (B) claims for opioids vs no opioid claims. Only variables that were significant in the bivariate analysis were included in the multivariate analysis but for variables with both continuous and categorical summary, only categorical variables were considered, to avoid multicollinearity. The reference category was the first alphabetically in each set of variables. Rheumatic disease (CCI comorbidity) was significant in the bivariate analysis but was discounted from the multivariate analysis (PPM vs no PPM) because of multicollinearity/low count. The following variables with low count (*n* < 3) have been omitted from the figure, none of which were significant: in the comparison of claims vs no claims for PPM (part A), payer channel other/unknown; mild liver disease (CCI morbidity); occupational therapy (pain-related healthcare service use); in the comparison of claims for opioids vs no claims (part B): payer channel other/unknown. OR = 1 indicates no association; OR > 1 suggests that the category is associated with a higher odds of the outcome compared with the reference category; OR < 1 suggests a lower odds. Statistically significant findings (*p* < .05) are indicated in bold. Findings trending towards significance are shown in italics*.* CCI, Charlson Comorbidity Index; CI, confidence interval; FFS, fee for service; OR, odds ratio; PPM, prescription pain medication; XLH, X-linked hypophosphatemia.

Multivariate analysis (Figure 2, panel A) revealed that the odds of claims for PPM (vs no claims) were double (odds ratio [OR] > 2.0) among patients aged ≥ 50 yr, those with Medicaid coverage, and those with myalgia, scoliosis, obesity, dental complications, or PT claims. Associations trended towards significance for arthralgia (1.80 [*p* = .088]) and spinal stenosis (2.20 [*p* = .054]).

For the comparison of adults who had claims for opioids vs claims for no opioids or PPM, the following variables were significant in the bivariate analysis (Tables S1 and S2): age and age group on the index date, geographic region, payer channel, CCI score, all the musculoskeletal conditions except kyphosis and scoliosis, all 3 non-musculoskeletal manifestations, “rickets,” vitamin D deficiency, dental complications, difficulty walking, muscle weakness, osteomalacia, claims for cholecalciferol, claims for PT or occupational therapy, and the number of PT visits. Race and metastatic solid tumor (a CCI-specific comorbidity) trended towards significance.

The multivariate analysis (Figure 2, panel B) revealed that the odds of claims for opioids vs no opioids were double (OR > 2.0) among those with claims for PT and those living in the South or West geographic regions vs the Midwest. Fracture and occupational therapy trended towards significance (OR 1.79 [*p* = .079] and 3.28 [*p* = .098], respectively).

#### Pediatric patients

**Figure 3:**
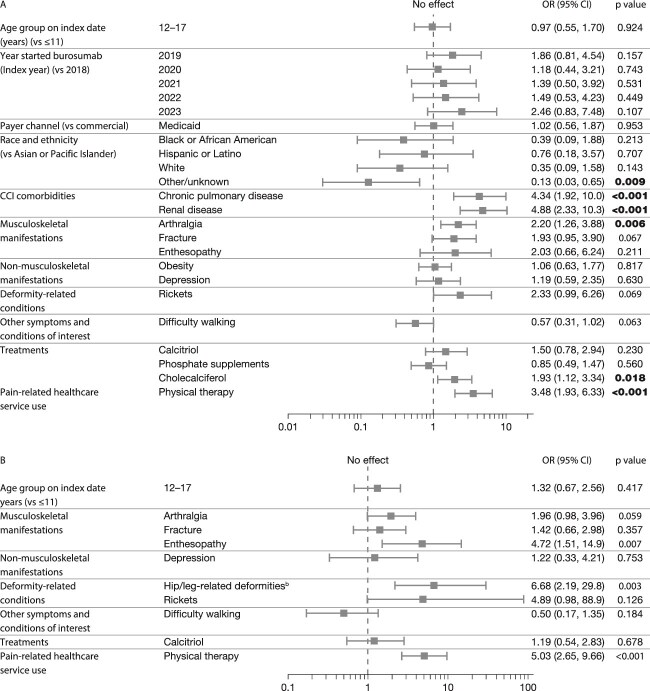
Results of the multivariate analysis comparing pediatric patients with XLH before burosumab treatment: (A) claims vs no claims for PPM; (B) claims for opioids vs no opioid claims. ^a^2024 was an incomplete year. ^b^Genu varum, genu valgum, varus deformities, and coxa vara. Only variables that were significant in the bivariate analysis were included in the multivariate analysis but for variables with both continuous and categorical summary, only categorical variables were considered, to avoid multicollinearity. The reference category was the first alphabetically in each set of variables. Genu varum (deformity-related condition) was significant in the bivariate analysis but was discounted from the multivariate analysis (PPM vs no PPM) because of multicollinearity. The following variables with low count (*n* < 3) have been omitted from the figure, none of which were significant: part A (claims vs no claims for PPM): index year 2024, payer channel Medicare Advantage/FFS and other/unknown; part B (claims for opioids vs no claims): any malignancy (CCI comorbidity). OR = 1 indicates no association; OR > 1 suggests that the category is associated with a higher odds of the outcome compared with the reference category; OR < 1 suggests a lower odds. Statistically significant findings (*p* < .05) are indicated in bold. Findings trending towards significance are shown in italics. CCI, Charlson Comorbidity Index; FFS, fee for service; OR, odds ratio; PPM, prescription pain medication; XLH, X-linked hypophosphatemia.

The following demographic variables were significant in the bivariate analysis comparing pediatric patients with and without claims for PPM (Table S3): age and age group on the index date, index year, and race. Several clinical variables were significant (Table S4): CCI score (and chronic pulmonary disease and renal disease among the CCI-specific comorbidities), arthralgia, fracture, and enthesopathy among the musculoskeletal conditions, depression and obesity among the non-musculoskeletal conditions, hip/leg-related deformities and rickets among the deformity-related conditions, and difficultly walking. Claims for other treatments (calcitriol, cholecalciferol, and phosphate supplements) and for PT were also significant. The multivariate analysis indicated that the odds of claims for PPM (vs no claims) among pediatric patients (Figure 3A) were lower in those with race other/unknown (vs Asian or Pacific Islander; OR 0.13) and were double (OR > 2) in those with chronic pulmonary disease, renal disease (both CCI-specific comorbidities), arthralgia, and claims for PT and slightly less than double in those with claims for cholecalciferol. Three factors trended towards significance: fracture (OR 1.93 [*p* = .067]), rickets (2.33 [*p* = .069]), and difficulty walking (0.57 [*p* = .063]).

For the comparison of pediatric patients making claims for opioids vs no opioids, the following variables were significant in the bivariate analysis (Tables S3 and S4): age and age group on the index date, CCI score (and any malignancy among the CCI-specific comorbidities), arthralgia, fracture, and enthesopathy among the musculoskeletal conditions, depression among the non-musculoskeletal conditions, hip/leg-related deformities and rickets among the deformity-related conditions, and difficulty walking. For other treatments, claims for calcitriol were a significant predictor, and claims for PT among the pain-related healthcare services. The multivariate analysis indicated that the odds of claims for opioids vs no opioids were more than 4 times higher in patients with claims for enthesopathy, hip/leg related deformities, and use of PT services (Figure 3B). Arthralgia trended towards significance (OR 1.96 [*p* = .059]).

### Changes in prescription pain medication claims after starting burosumab

#### Adults

A total of 288 adults were continuously enrolled for medical and pharmacy benefits during the baseline period (the 12 mo before the first burosumab claim) and the subsequent 12 mo, and 214 for the baseline period and the subsequent 24 mo. Patient and clinical characteristics are provided in Tables S5 and S6. Changes in PPM claims from baseline for each follow-up period are shown in Table 3. Considering the cohort with 12 mo’ follow-up data, 53% had claims for any PPM during baseline and 38% had claims for opioids. There was little change in PPM claims during follow-up: 49% had claims for any PPM and 34% for opioids. For the cohort of 153 adults who had claims for any PPM during baseline, 67% had claims for any PPM during follow-up—a marked decrease. In this cohort, the proportion making claims for opioids decreased from 71% to 48%. Of the 108 patients who had claims for opioids during baseline, 60% had claims for opioids during follow-up, and 72% had claims for any PPM. Nearly half of the adults (*n* = 135) made no claims for PPM during baseline. Of these, 29% subsequently had claims for any PPM during follow-up (19% for opioids). There was little change in the proportions of patients accessing pain-related healthcare services across all groups. Results were similar for the cohort with 24 mo’ follow-up data (Table 3).

**Table 3:** Change from baseline in claims in adults for PPM and pain-related healthcare services in the 12 and 24 mo following the first burosumab claim, according to claims for PPM during baseline.

**Group based on claims during BL**	***n***	**Time period**	**PPM claims during follow-up**		**Claims for pain-related healthcare services during follow-up**		
			**Any**	**Any opioid** ^**a**^	**Physical therapy**	**Occupational therapy**	**Alternative medicine** ^**b**^
**12 mo’ follow-up, *n* (%)**							
** All patients**	288	BL	153 (53.1)	108 (37.5)	72 (25.0)	16 (5.6)	20 (6.9)
		Follow-up	142 (49.3)	99 (34.4)	74 (25.7)	13 (4.5)	16 (5.6)
** Patients with claims for any PPM during BL**	153	BL	153 (100.0)	108 (70.6)	52 (34.0)	14 (9.2)	12 (7.8)
		Follow-up	103 (67.3)	74 (48.4)	46 (30.1)	7 (4.6)	7 (4.6)
** Patients with claims for opioids^a^ during BL**	108	BL	108 (100.0)	108 (100.0)	42 (38.9)	12 (11.1)	7 (6.5)
		Follow-up	78 (72.2)	65 (60.2)	38 (35.2)	6 (5.6)	6 (5.6)
** Patients with no PPM claims during BL**	135	BL	0 (0.0)	0 (0.0)	20 (14.8)	≤3	8 (5.9)
		Follow-up	39 (28.9)	25 (18.5)	28 (20.7)	6 (4.4)	9 (6.7)
**24 mo’ follow-up, *n* (%)**							
** All patients**	214	BL	109 (50.9)	74 (34.6)	56 (26.2)	14 (6.5)	18 (8.4)
		Follow-up	103 (48.1)	74 (34.6)	48 (22.4)	15 (7.0)	17 (7.9)
** Patients with claims for any PPM during BL**	109	BL	109 (100.0)	74 (67.9)	38 (34.9)	12 (11.0)	11 (10.1)
		Follow-up	69 (63.3)	54 (49.5)	28 (25.7)	10 (9.2)	9 (8.3)
** Patients with claims for opioids^a^ during BL**	74	BL	74 (100.0)	74 (100.0)	30 (40.5)	11 (14.9)	6 (8.1)
		Follow-up	52 (70.3)	45 (60.8)	21 (28.4)	6 (8.1)	7 (9.5)
** Patients with no PPM claims during BL**	105	BL	0 (0.0)	0 (0.0)	18 (17.1)	≤3	7 (6.7)
		Follow-up	34 (32.4)	20 (19.0)	20 (19.0)	5 (4.8)	8 (7.6)

Abbreviations: BL = baseline; HIPAA = Health Insurance Portability and Accountability Act; PPM = prescription pain medication.

a Patients with claims for opioids could have also have claims for non-opioids.

b Acupuncture and chiropractic services.

Counts ≤3 were masked to comply with HIPAA regulations.

BL refers to the 12 mo before the first burosumab claims; follow-up was for the subsequent 12 or 24 mo.

Opioid prescriptions covered a mean of 99 d during baseline, increasing to 133 during the 12 mo’ follow-up and changing from 109 at baseline to 120 during the 24 mo’ follow-up; however, SDs are large, indicating wide variability (Table S7). The mean daily dose of opioids (in morphine equivalents) decreased from 1235 mg during baseline to 869 mg during the 12 mo’ follow-up, and it decreased from 1197 mg at baseline to 986 mg in during the 24 mo follow-up period. However, the SDs are also large, indicating wide variability, and the average daily dose was driven by 1 patient receiving a very high dose of oxycodone.

#### Pediatric patients

A total of 412 pediatric patients were continuously enrolled for medical and pharmacy benefits during the baseline period and the subsequent 12 mo, and 322 for the baseline period and the subsequent 24 mo. Patient and clinical characteristics are provided in Tables S8 and S9. Changes in PPM claims from baseline for each follow-up period are shown in Table 4. Considering the cohort with 12 mo’ follow-up, 22% had claims for any PPM during baseline and 12% had claims for opioids, with little change in the proportion of patients with claims for any PPM during the 12 mo’ follow-up (23%), but with a slight increase in claims for opioids (17%). Among the 89 patients who had any claims for any PPM during baseline, 42% had claims for any PPM during follow-up, but the proportion making claims for opioids decreased from 57% to 32%. Among the 51 patients who had claims for opioid prescriptions during baseline, 41% had claims for opioids during follow-up, and 41% had claims for any PPM. Over three-quarters (78%) made no claims for PPM during baseline. Of these, 18% subsequently had claims for any PPM during follow-up (13% for opioids). There was little change in the proportions of patients accessing pain-related healthcare services across most groups, other than decreases in claims for PT in patients with any PPM claim during baseline (38% to 27%) and in those with claims for opioids during baseline (53% to 37%).

**Table 4:** Change from baseline in claims in pediatric patients for PPM and pain-related healthcare services in the 12 and 24 mo following the first burosumab claim.

		**Time period**	**PPM claims during follow-up**		**Pain-related healthcare services during follow-up**		
			**Any**	**Any opioid^**a**^**	**Physical therapy**	**Occupational therapy**	**Alternative medicine^**b**^**
**12 mo’ follow-up, *n* (%)**							
** All patients**	412	BL	89 (21.6)	51 (12.4)	76 (18.4)	18 (4.4)	5 (1.2)
		Follow-up	96 (23.3)	69 (16.7)	63 (15.3)	12 (2.9)	5 (1.2)
** Patients with claims for any PPM during BL**	89	BL	89 (100.0)	51 (57.3)	34 (38.2)	5 (5.6)	≤3
		Follow-up	37 (41.6)	28 (31.5)	24 (27.0)	≤3	≤3
** Patients with claims for opioids^**a**^ during BL**	51	BL	51 (100.0)	51 (100.0)	27 (52.9)	≤3	≤3
		Follow-up	21 (41.2)	21 (41.2)	19 (37.3)	≤3	≤3
** Patients with no PPM claims during BL**	323	BL	0 (0.0)	0 (0.0)	42 (13.0)	13 (4.0)	4 (1.2)
		Follow-up	59 (18.3)	41 (12.7)	39 (12.1)	9 (2.8)	≤3
**24 mo’ follow-up, *n* (%)**							
** All patients**	322	BL	79 (24.5)	47 (14.6)	65 (20.2)	13 (4.0)	≤3
		Follow-up	52 (16.1)	40 (12.4)	45 (14.0)	12 (3.7)	10 (3.1)
** Patients with claims for any PPM during BL**	79	BL	79 (100.0)	47 (59.5)	34 (43.0)	5 (6.3)	≤3
		Follow-up	15 (19.0)	11 (13.9)	16 (20.3)	≤3	5 (6.3)
** Patients with claims for opioids^**a**^ during BL**	47	BL	47 (100.0)	47 (100.0)	27 (57.4)	≤3	≤3
		Follow-up	7 (14.9)	7 (14.9)	10 (21.3)	≤3	4 (8.5)
** Patients with no PPM claims during BL**	243	BL	0 (0.0)	0 (0.0)	31 (12.8)	8 (3.3)	≤3
		Follow-up	37 (15.2)	29 (11.9)	29 (11.9)	9 (3.7)	5 (2.1)

Abbreviations: BL, baseline; HIPAA, Health Insurance Portability and Accountability Act; PPM, prescription pain medication.

a Patients with any claims for opioids could have also have claims for non-opioids.

b Acupuncture and chiropractic services.

Counts ≤3 were masked to comply with HIPAA regulations.

BL refers to the 12 mo before the first burosumab claims; follow-up was for the subsequent 12 or 24 mo.

In contrast to the adult cohort, claims for PPM decreased from baseline to the 24 mo’ follow-up period in pediatric patients, from 25% to 16% for any PPM and from 15% to 12% for opioids. While similar proportions had claims for any PPM (25%) or opioids (15%) to the patients with 12 mo’ follow-up data, among the cohort of 79 pediatric patients who had any claims for any PPM during baseline, only 19% had claims for any PPM during follow-up, and the proportion making claims for opioids decreased from 60% to 14%. Of the 47 patients who had claims for opioid prescriptions during baseline, only 15% had claims for opioids during follow-up, and 15% had claims for any PPM. Claims for PT decreased in all groups.

The number of days covered by opioid prescriptions was 10-fold lower in the pediatric patients than in adults and changed little after burosumab (Table S7). The mean daily dose of opioids was also lower than in adults and changed little during burosumab treatment.

## Discussion

This study describes the demographic and clinical characteristics of relatively large numbers of patients with XLH in the US real-world practice, identifies characteristics that may be associated with the use of PPM, and provides an early evaluation of the effect of burosumab treatment on PPM use. Our study provides a novel and broader perspective on pain in XLH, as the limited studies to date have been small and/or qualitative, and we believe that this is the first study to consider adults and pediatric patients separately and to explore factors associated with the use of PPM in patients with XLH.

Clinical characteristics were markedly different between pediatric patients and adults with XLH in this analysis, indicating a substantial and accumulating disease burden, which is consistent with the known progressive musculoskeletal involvement in XLH. An earlier study using the same data source (2015-2022) similarly reported a substantial disease burden in 1358 patients (53% children) with XLH (identified using the same criteria as the current study) before burosumab treatment.^11^ The adult cohort in the current study had a notable prevalence of musculoskeletal and deformity-related conditions (likely acquired during childhood), dental complications, tinnitus, and obesity, all classic sequelae of XLH. However, only conditions for which patients had made insurance claims are included, so the frequency of comorbidities is likely to be higher in clinical practice. Nevertheless, this burden profile is consistent with other reports in adults with XLH.^6^^,^^14^^,^^28^ A substantial proportion of the adult cohort had claims for calcitriol (66%) and phosphate supplements (42%), indicating persistent hypophosphatemia, which may contribute to pain.

Just over half of the adult cohort (54%) made claims for PPM during the 12 mo before starting burosumab treatment; 38% made claims for opioids (68% of patients with any PPM claim), suggesting more severe pain. One-quarter had claims for PT, with a frequency of every 3-4 wk. Just less than half did not make any claims for PPM during baseline but may have been taking over-the-counter products.

The multivariate analysis (Figure 2) indicated that older age, the Medicaid payer channel, musculoskeletal conditions (myalgia and scoliosis), dental complications, obesity, and claims for PT were associated with claims for PPM in adults, and arthralgia and spinal stenosis trended towards significance. Older patients are more likely to have accumulated more musculoskeletal complications that cause pain and more dental complications than younger patients. Other musculoskeletal manifestations that were not significant in the multivariate model are, nevertheless, likely to cause pain in some patients, such as osteoarthritis and enthesopathy.

Enthesopathy is an important cause of pain in adults with XLH. The frequency of enthesopathy was lower in this study than reported in the literature. For example, a full-body imaging study of 9 adults with XLH identified extensive enthesopathies of the upper body, lower body, and spine,^29^ and another imaging study of 25 adults with XLH reported that up to 96% had enthesopathies of the hip, 72% in the Achilles tendon, and 68% in the knee joint,^30^ compared with 29% in the current study. This disparity may reflect a lack of documented comprehensive imaging in real-world practice. In addition, the current analysis reports claims related to enthesopathy, which may contribute to the low apparent frequency (eg, if patients with enthesopathies have not made claims with that code). The pathophysiology of enthesopathies in XLH is not fully understood.^31^ An international panel of XLH experts concluded that, while enthesopathies are unlikely to be reversed by burosumab treatment, they may be prevented or their progression slowed^32^; this may at least partly explain why a decrease in PPM claims was not seen in adults with XLH receiving burosumab.

Obesity is a known complication of XLH^33^ and may reflect short stature (ie, more severe disease). While it may be a consequence of impaired mobility/physical activity, obesity may also cause pain due to pressure on deformed joints. Although depression was not associated with greater PPM use, there is an established, and complicated, interrelationship between chronic pain and depression.^34^^,^^35^ Claims for opioids were not related to musculoskeletal manifestations of XLH, deformity-related conditions, or other known complications, although the association with fractures trended towards significance. The relationship with PT (and the association with occupational therapy trending towards significance, likely reflecting the small number of claims) may indicate that patients with more severe pain require several approaches to pain management. Tailored PT programs have been shown to improve functional ability and reduce pain in patients with XLH.^36^ Routine dental treatment is not covered under medical insurance; claims in the current study are, therefore, likely to reflect more severe dental issues, another long-term manifestation of XLH. In this study, claims for dental treatment were more frequent among the pediatric population.

The pediatric cohort was mostly younger children (≤11 yr) (65%). They had fewer claims for musculoskeletal conditions than adults, as would be expected given the progressive nature of XLH, although a notable proportion (39%) had claims for arthralgia. Claims for depression, hypertension, and obesity were also lower in pediatric patients, consistent with the overall population demographic.^37–39^ However, claims for deformity-related conditions—notably rickets, anatomical leg and hip deformities, and short stature—were higher in pediatric patients, likely because these conditions develop during childhood as the bones begin to bear weight, whereas hip deformities in adults are more likely to be related to degenerative osteoarthritis, osteophytes, and enthesopathy, and the accumulation of progressive comorbid conditions. Claims for delayed walking/growth and difficulty walking were also higher in pediatric patients.

About one-quarter of the pediatric cohort (23%) made claims for PPM during the baseline period, which is consistent with the lower frequency of musculoskeletal manifestations in younger patients. For those who did make PPM claims, slightly more were for opioids (± non-opioids) than for no opioids. In the multivariate analysis, claims for any PPM were associated with race, chronic pulmonary disease, and renal disease among the CCI comorbidities, arthralgia among the musculoskeletal manifestations of XLH (which was not the case in adults), cholecalciferol treatment, and PT. Associations with fracture, rickets, and difficulty walking trended towards significance. Thus, many of the manifestations and consequences of XLH are related to PPM use in children. The association between pain use and chronic pulmonary disease was unexpected, as this is not a known complication of XLH. However, children with osteogenesis imperfecta may have similar anatomical restrictions to children with XLH; in the former, these anatomical changes have been associated with restrictive lung function.^40^ Claims for opioids were significantly associated with enthesopathy among the musculoskeletal manifestations (and trended towards significance for arthralgia), leg/hip-related deformities, and PT. The combination of PT and stronger pain medication may indicate more severe disease or may relate to surgery—many children with XLH undergo surgery to correct genu varum/valgum deformities and are likely to be prescribed opioids and PT. The association with enthesopathy in pediatric patients is unexpected, as enthesopathies are rare in children; this may indicate more severe disease or may reflect inaccurate coding.

The stark contrast in the number of claims for PPM between adults and pediatric patients is consistent with the progressive nature of XLH and the development of potentially painful musculoskeletal conditions due to chronic hypophosphatemia. Our findings are consistent with other studies reporting a high incidence of pain in patients with XLH.^6^^,^^8^^,^^11^ Bone pain has been linked to osteomalacia and joint pain to osteoarthritis from weight bearing on misaligned joints in adulthood.^6^ Thus, preventing disease progression would be expected to prevent the development of painful comorbidities.

Given that the pain of XLH is multifaceted and variable,^8^^,^^16^ patients’ requirements for pain management are also likely to vary. A substantial proportion of adults made PPM claims during baseline (54%). This is consistent with clinical studies reporting high use of pain medication.^6^^,^^16^ That some patients did not make claims for PPM in the current analysis likely reflects the variability and episodic nature of pain associated with XLH, and that, at times, pain may be adequately managed with nonprescription medication.^6^ Prescribing practices may also have an influence: clinicians may be hesitant to prescribe opioids because of concerns about sedation and other side-effects, misuse, and dependency; prescribing controlled substances also has an administrative burden. Pain may be underreported in children, and prescribers and parents/carers may be reluctant to prescribe opioids. In addition, pediatric patients lack autonomy in the approach to pain management. In adults, claims for PPM were associated with Medicaid coverage. This may reflect an impact of XLH-related disability on income and access to insurance/healthcare. A recent real-world study reported that restrictions in physical functioning associated with XLH impacted patients’ work productivity.^15^

The effectiveness of pain medicines can be highly variable and may reduce but not entirely relieve pain.^8^ Patients may also use nonpharmacological strategies to manage XLH-related pain, such as PT, massage, exercise and stretching, lifestyle adjustments, yoga, acupuncture, and assistive devices.^8^^,^^16^ In the current analysis, claims for PT were associated with the use of PPM but we cannot know whether patients were also paying for other therapies out-of-pocket.

This study also explored changes in the number of claims for PPM overall and for opioids, after 12 or 24 mo of burosumab treatment. Based on its mechanism of action, burosumab is not expected to provide pain relief directly (ie, an analgesic effect); however, improvements in skeletal symptoms such as bone mineralization and healing of fractures/pseudofractures, and rickets in children, may be expected to improve pain indirectly. Patients in clinical trials^19^^,^^22^^,^^23^^,^^41^^,^^42^ and real-world studies^26^ have reported improvements in self-reported pain during burosumab treatment, from as early as 24 wk in adults.^19^ In the current study, the proportion of patients making claims for PPM during baseline was higher in the adult than in the pediatric cohort. There was little change in PPM claims in the overall adult population, although among those making claims for PPM during baseline, claims for any PPM and for opioids decreased during follow-up (Table 3). In the pediatric cohort, claims for PPM decreased from baseline at the 24 mo’ follow-up (Table 4). The number of days covered by opioid prescriptions was much lower than in adults, which may reflect short-term use following surgery, for example. The apparent decrease in PPM claims is consistent with reductions in patient-reported pain scores with burosumab treatment reported in clinical trials.^19^^,^^42^

The extent to which the painful manifestations of XLH are modifiable with burosumab treatment differs between children and adults. In children, improvements in bone structure with treatment may reduce the frequency of painful sequelae. In adults, skeletal morbidities acquired in childhood and degenerative conditions such as osteoarthritis cannot be reversed and may lead to persistent pain. However, burosumab may still prevent the development of further potentially painful skeletal manifestations (eg, enthesopathy and fracture), improve osteomalacia, and heal fractures and pseudofractures,^32^ and it has been associated with a reduced incidence of dental complications.^43^ The current study does not record the severity of pain. The Phase 3 clinical trial of burosumab^21^ required patients to have moderately severe pain, whereas patients in the real-world setting will have highly variable pain, so the degree of pain relief is also likely to vary. A real-world pain study in adults with XLH reported significant improvements in patient-reported pain scores after 12 mo’ burosumab treatment compared with active vitamin D and phosphate supplements.^44^ Another recent real-world study also reported that self-reported pain scores decreased during burosumab treatment, but the proportion of patients taking pain medication did not change.^26^ However, a reduction in pain scores may not necessarily mean the absence of pain, and patients may continue to have some pain that requires medication.

Decreased use of both non-opioid and opioid pain medication is likely to be beneficial for patients given the potential side-effects associated with non-steroidal anti-inflammatory drugs and the risk of tolerance, dependence, and overdose with opioids. There is also considerable debate and concern about the use of opioids in the treatment of chronic pain.^45^^,^^46^

A strength of this study is its use of real-world data from a relatively large number of patients (given that XLH is a rare disease) of different ages and with XLH symptoms of variable severity. Whereas clinical trials may be restricted to patients with more severe XLH, for example based on pain scores, the current study included all patients with familial hypophosphatemia who were prescribed burosumab and were, therefore, assumed to have XLH. It is possible, but unlikely given the indication for burosumab, that patients with other hypophosphatemias were included. However, patients with XLH who were not prescribed burosumab will not have been identified, although these are likely to have less severe XLH symptoms, and burosumab is largely prescribed for patients with more severe symptoms.^44^ Thus, the sample may not be representative of the real-world demographic or the use of pain medication in adults with less severe XLH.

A limitation of this study is that claims are based on ICD-10 codes, not necessarily the diagnosis given to the patient. For example, several ICD-10 codes relating to rickets are not age-restricted and may appear in adult claims data, such as E83.31 (vitamin D-resistant rickets), E55.0 (active rickets [nutritional/vitamin D deficiency]) and E64.3 (sequelae [inactive] rickets]) and may represent sequelae from prior disease, persistence of childhood disease manifestations, late diagnosis, or miscoding. In addition, only comorbidities that are related to an insurance claim were considered, which may have led to the underestimation of some conditions, and some associations between comorbidities and PPM may have been missed. The use of non-PPM and services paid for out-of-pocket is not known, so the overall picture of pain management is incomplete. Furthermore, the reason for requesting PPM (ie, for XLH or another unrelated condition) is not recorded, nor is the duration of use. As pain levels are not recorded, the effectiveness of pain relief is not known, nor is adherence to pain medication. Claims database analyses are subject to the limitations of the data recorded, and misclassification of diagnoses and outcomes may occur. While this is a relatively large study in a rare disease, the statistical power is nevertheless limited. In addition, many patients will have multiple manifestations of XLH, meaning that some granularity in the model is lost when adjusting for multicollinearity. The data collection period after burosumab treatment was relatively short; while some reduction in pain medication use was seen over the first 2 yr of burosumab treatment, a longer real-world study specifically exploring relationships between pain scores, PPM use (prescribed and over-the-counter), and XLH symptoms would provide greater insight.

## Conclusion

Overall PPM use was lower in pediatric patients than in adults, and factors associated with PPM claims (and opioid claims) differed between the 2 populations. This is consistent with the higher frequency of symptoms and longer-term manifestations of XLH in adults. Claims for PPM may reflect more severe XLH or greater engagement with the healthcare system, which may explain the association between PPM use and the use of other pain-related services such as PT. Reduced claims for PPM after 24 mo’ burosumab treatment are consistent with real-world studies reporting decreases in self-reported pain during burosumab treatment. A longer real-world study would help define the causes and age-specific features of XLH-related pain; assessing how burosumab influences pain over time would guide more patient-centered approaches to pain management.

## Supplementary Material

XLH_pain_management_supplementary_resubmission_05MAR26_ziag047

## Data Availability

Data supporting the findings of this study are available from the corresponding author upon reasonable request.
