# Supplementary material for: Pain medication use in patients with X-linked hypophosphatemia treated with burosumab: a retrospective real-world study
Source: JBMR Plus. 2026 Mar 24;10(5):ziag047. doi: 10.1093/jbmrpl/ziag047 (PMC13117624; doi:10.1093/jbmrpl/ziag047)
Supplement: XLH_pain_management_supplementary_resubmission_05MAR26_ziag047 [file xlh_pain_management_supplementary_resubmission_05mar26_ziag047.docx]

# Supplementary tables

Supplementary Table 1. Bivariate analysis: baseline (pre-treatment) demographic characteristics of adults (≥18 years) with XLH who received burosumab treatment according to PPM claims during baseline (PPM vs no PPM; opioid vs no opioid)

|  | PPM  (n=208) | No PPM  (n=179) | p value | Opioids^a^  (n=146) | No opioids  (n=241) | p value |
| --- | --- | --- | --- | --- | --- | --- |
| **Age on index date, years, mean ± SD** | 40.6 ± 14.9 | 34.6 ± 14.2 | **<0.001** | 41.3 ± 14.6 | 35.7 ± 14.7 | **<0.001** |
| Age group on index date (years), n (%) | | | | | | |
| 18–29 | 58 (27.9) | 80 (44.7) | **0.001** | 38 (26.0) | 100 (41.5) | **0.006** |
| 30–39 | 42 (20.2) | 36 (20.1) |  | 27 (18.5) | 51 (21.2) |  |
| 40–49 | 48 (23.1) | 36 (20.1) |  | 38 (26.0) | 46 (19.1) |  |
| ≥50 | 60 (28.8) | 27 (15.1) |  | 43 (29.5) | 44 (18.3) |  |
| Sex | | | | | | |
| Female | 147 (70.7%) | 116 (64.8%) | 0.368 | 105 (71.9) | 158 (65.6) | 0.425 |
| Male | 59 (28.4%) | 59 (33.0%) |  | 39 (26.7) | 79 (32.8) |  |
| Unknown | <3 | 4 (2.2%) |  | <3 | 4 (1.7) |  |
| Year started burosumab (index year), n (%) | | | | | | |
| 2018 | 16 (7.7) | 13 (7.3) | 0.677 | 11 (7.5) | 18 (7.5) | 0.480 |
| 2019 | 62 (29.8) | 49 (27.4) |  | 44 (30.1) | 67 (27.8) |  |
| 2020 | 40 (19.2) | 29 (16.2) |  | 26 (17.8) | 43 (17.8) |  |
| 2021 | 37 (17.8) | 27 (15.1) |  | 28 (19.2) | 36 (14.9) |  |
| 2022 | 29 (13.9) | 32 (17.9) |  | 20 (13.7) | 41 (17.0) |  |
| 2023 | 20 (9.6) | 22 (12.3) |  | 16 (11.0) | 26 (10.8) |  |
| 2024 | 4 (1.9) | 7 (3.9) |  | <3 | 10 (4.1) |  |
| Geographic region, n (%) | | | | | | |
| Midwest | 48 (23.1) | 52 (29.1) | 0.309 | 34 (23.3) | 66 (27.4) | **0.024** |
| Northeast | 39 (18.8) | 41 (22.9) |  | 22 (15.1) | 58 (24.1) |  |
| South | 84 (40.4) | 59 (33.0) |  | 59 (40.4) | 84 (34.9) |  |
| West | 36 (17.3) | 27 (15.1) |  | 30 (20.5) | 33 (13.7) |  |
| Unknown | <3 | 0 (0.0) |  | <3 | 0 (0.0) |  |
| Payer channel, n (%) | | | | | | |
| Commercial | 115 (55.3) | 123 (68.7) | **0.016** | 78 (53.4) | 160 (66.4) | **0.013** |
| Medicaid | 64 (30.8) | 41 (22.9) |  | 43 (29.5) | 62 (25.7) |  |
| Medicare Advantage/FFS | 28 (13.5) | 13 (7.3) |  | 24 (16.4) | 17 (7.1) |  |
| Other/unknown | <3 | <3 |  | <3 | <3 |  |
| Race, n (%) | | | | | | |
| Asian or Pacific Islander | <3 | 7 (3.9) | 0.051 | <3 | 7 (2.9) | 0.053 |
| Black or African American | 22 (10.6) | 12 (6.7) |  | 16 (11.0) | 18 (7.5) |  |
| Hispanic or Latino | 25 (12.0) | 19 (10.6) |  | 19 (13.0) | 25 (10.4) |  |
| White | 116 (55.8) | 90 (50.3) |  | 84 (57.5) | 122 (50.6) |  |
| Other/unknown | 44 (21.2) | 51 (28.5) |  | 26 (17.8) | 69 (28.6) |  |
| ^a^Patients with claims for opioids could also have claims for non-opioids.  Wilcoxon rank sum test; Pearson’s Chi-squared test with simulated P value (based on 2000 replicates); statistically significant findings (p<0.05) indicated in **bold**.  Payer channel categories were mutually exclusive.  Counts <3 have been masked to comply with HIPAA regulations.  FFS, fee for service; HIPAA, Health Insurance Portability and Accountability Act; PPM, prescription pain medication; SD, standard deviation; XLH, X-linked hypophosphatemia. | | | | | | |

Supplementary Table 2. Bivariate analysis: baseline (pre-treatment) clinical characteristics of adults (≥18 years) with XLH who received burosumab according to PPM claims during baseline (PPM vs no PPM; opioid vs no opioid)

|  | | PPM  (n=208) | No PPM  (n=179) | P value | Opioids^a^  (n=146) | No opioids  (n=241) | P value |
| --- | --- | --- | --- | --- | --- | --- | --- |
| **CCI score, mean ± SD** | | 0.7 ± 1.3 | 0.3 ± 0.7 | **<0.001** | 0.7 ± 1.4 | 0.3 ± 0.8 | **<0.001** |
| CCI comorbidities, n (%)^b^ | | | | | | | |
| Any malignancy | | 10 (4.8) | 4 (2.2) | 0.269 | 8 (5.5) | 6 (2.5) | 0.157 |
| Chronic pulmonary disease | | 33 (15.9) | 22 (12.3) | 0.388 | 27 (18.5) | 28 (11.6) | 0.072 |
| Diabetes without chronic complication | | 16 (7.7) | 5 (2.8) | **0.036** | 10 (6.8) | 11 (4.6) | 0.338 |
| Metastatic solid tumor | | 3 (1.4) | 0 (0.0) | 0.245 | 3 (2.1) | 0 (0.0) | 0.057 |
| Mild liver disease | | 9 (4.3) | <3 | **0.018** | 6 (4.1) | 4 (1.7) | 0.187 |
| Renal disease | | 18 (8.7) | 8 (4.5) | 0.109 | 14 (9.6) | 12 (5.0) | 0.091 |
| Rheumatic disease | | 11 (5.3) | 0 (0.0) | **<0.001** | 7 (4.8) | 4 (1.7) | 0.105 |
| Musculoskeletal manifestations, n (%) | | | | | | | |
| Osteoarthritis | | 148 (71.2) | 69 (38.5) | **<0.001** | 112 (76.7) | 105 (43.6) | **<0.001** |
| Arthralgia | | 183 (88.0) | 109 (60.9) | **<0.001** | 130 (89.0) | 162 (67.2) | **<0.001** |
| Fracture | | 81 (38.9) | 34 (19.0) | **<0.001** | 60 (41.1) | 55 (22.8) | **<0.001** |
| Myalgia | | 73 (35.1) | 21 (11.7) | **<0.001** | 56 (38.4) | 38 (15.8) | **<0.001** |
| Spinal stenosis | | 68 (32.7) | 14 (7.8) | **<0.001** | 53 (36.3) | 29 (12.0) | **<0.001** |
| Enthesopathy | | 75 (36.1) | 36 (20.1) | **<0.001** | 53 (36.3) | 58 (24.1) | **0.012** |
| Kyphosis | | 7 (3.4) | 1 (0.6) | 0.075 | 5 (3.4) | 3 (1.2) | 0.294 |
| Scoliosis | | 30 (14.4) | 9 (5.0) | **0.003** | 17 (11.6) | 22 (9.1) | 0.485 |
| Non-musculoskeletal manifestations, n (%) | | | | | | | |
| Hypertension | | 120 (57.7) | 59 (33.0) | **<0.001** | 80 (54.8) | 99 (41.1) | **0.009** |
| Obesity | | 156 (75.0) | 92 (51.4) | **<0.001** | 112 (76.7) | 136 (56.4) | **<0.001** |
| Depression | | 110 (52.9) | 58 (32.4) | **<0.001** | 80 (54.8) | 88 (36.5) | **0.001** |
| Deformity-related conditions, n (%) | | | | | | | |
| Chiari malformation | | 9 (4.3) | 9 (5.0) | 0.799 | 7 (4.8) | 11 (4.6) | >0.999 |
| Craniosynostosis | | 0 (0.0) | <3 | 0.448 | 0 (0.0) | <3 | >0.999 |
| Delayed growth/delayed walking | | 0 (0.0) | <3 | 0.215 | 0 (0.0) | <3 | 0.516 |
| Short stature | | 16 (7.7) | 14 (7.8) | >0.999 | 11 (7.5) | 19 (7.9) | >0.999 |
| Hip/leg-related deformities^c^ | | 40 (19.2) | 35 (19.6) | >0.999 | 26 (17.8) | 49 (20.3) | 0.589 |
| Rickets^d^ | | 153 (73.6) | 96 (53.6) | **<0.001** | 105 (71.9) | 144 (59.8) | **0.016** |
| Other symptoms and conditions of interest, n (%) | | | | | | | |
| Vitamin D deficiency | | 140 (67.3) | 108 (60.3) | 0.168 | 104 (71.2) | 144 (59.8) | **0.024** |
| Dental complications | | 50 (24.0) | 21 (11.7) | **0.002** | 35 (24.0) | 36 (14.9) | **0.034** |
| Difficulty walking | | 78 (37.5) | 32 (17.9) | **<0.001** | 60 (41.1) | 50 (20.7) | **<0.001** |
| Muscle weakness | | 51 (24.5) | 23 (12.8) | **0.004** | 42 (28.8) | 32 (13.3) | **<0.001** |
| Hearing loss | | 62 (29.8) | 39 (21.8) | 0.082 | 45 (30.8) | 56 (23.2) | 0.131 |
| Hyperparathyroidism | | 61 (29.3) | 38 (21.2) | 0.086 | 44 (30.1) | 55 (22.8) | 0.115 |
| Kidney stone | | 29 (13.9) | 15 (8.4) | 0.102 | 20 (13.7) | 24 (10.0) | 0.330 |
| Nephrocalcinosis | | 20 (9.6) | 22 (12.3) | 0.430 | 12 (8.2) | 30 (12.4) | 0.232 |
| Osteomalacia | | 41 (19.7) | 25 (14.0) | 0.140 | 34 (23.3) | 32 (13.3) | **0.012** |
| Tinnitus | | 31 (14.9) | 17 (9.5) | 0.118 | 22 (15.1) | 26 (10.8) | 0.274 |
| Vertigo | | 11 (5.3) | 4 (2.2) | 0.193 | 7 (4.8) | 8 (3.3) | 0.591 |
| Treatments, n (%) | | | | | | | |
| Phosphate salts/active vitamin D | Calcitriol | 141 (67.8) | 114 (63.7) | 0.470 | 96 (65.8) | 159 (66.0) | >0.999 |
|  | Phosphate supplements | 90 (43.3) | 71 (39.7) | 0.531 | 62 (42.5) | 99 (41.1) | 0.825 |
| Other conventional vitamin D | Cholecalciferol | 36 (17.3) | 19 (10.6) | 0.075 | 28 (19.2) | 27 (11.2) | **0.036** |
| Pain-related healthcare service utilization | | | | | | | |
| Physical therapy | Patients with visits, n (%) | 74 (35.6) | 23 (12.8) | **<0.001** | 60 (41.1) | 37 (15.4) | **<0.001** |
|  | Number of visits, mean ± SD | 15.3 ± 14.1 | 11.1 ± 13.2 | **0.047** | 16.4 ± 14.8 | 10.9 ± 11.7 | **0.021** |
| Occupational therapy | Patients with visits, n (%) | 17 (8.2) | <3 | **<0.001** | 15 (10.3) | 4 (1.7) | **<0.001** |
|  | Number of visits, mean ± SD | 3.8 ± 7.5 | 1.0 ± 0.0 | 0.317 | 4.2 ± 7.9 | 1.0 ± 0.0 | 0.118 |
| Alternative medicine^e^ | Patients with visits, n (%) | 20 (9.6) | 9 (5.0) | 0.127 | 14 (9.6) | 15 (6.2) | 0.256 |
|  | Number of visits, mean ± SD | 11.1 ± 10.8 | 14.0 ± 12.8 | 0.722 | 12.4 ± 11.8 | 11.6 ± 11.3 | 0.676 |
| ^a^Patients with claims for opioids could also have claims for non-opioids.  ^b^CCI comorbidities with claims by ≥5% of patients in either age group, or that were significant, are reported.  ^c^Genu varum, genu valgum, varus deformities, and coxa vara.  ^d^Several diagnostic codes relating to disorders of bone mineralization are not age-restricted and may appear in adult claims data, such as E83.31 (Vitamin D-resistant rickets), E55.0 (active rickets [nutritional/vitamin D deficiency]) and E64.3 (sequelae [inactive] rickets]). These may represent sequelae from prior disease, persistence of childhood disease manifestations, late diagnosis or miscoding.  ^e^Acupuncture and chiropractic services.  Clinical characteristics were assessed during baseline (the 12 months before the first prescription for burosumab [the index date]) for patients with continuous medical and pharmacy benefits during that period.  Wilcoxon rank sum test; Pearson’s Chi-squared test with simulated P value (based on 2000 replicates); statistically significant findings (p<0.05) indicated in **bold**.  Counts <3 were masked to comply with HIPAA regulations.  CCI, Charlson Comorbidity Index; HIPAA, Health Insurance Portability and Accountability Act; PPM, prescription pain medication; SD, standard deviation; XLH, X-linked hypophosphatemia. | | | | | | | |

Supplementary Table 3. Bivariate analysis: baseline (pre-treatment) demographic characteristics of pediatric patients (<18 years) with XLH who received burosumab treatment according to PPM claims during baseline (PPM vs no PPM; opioid vs no opioid)

|  | PPM  (n=123) | No PPM  (n=401) | P value | Opioids^a^  (n=65) | No opioids  (n=459) | P value |
| --- | --- | --- | --- | --- | --- | --- |
| **Age on index date,** mean ± SD | 10.5 ± 4.9 | 8.4 ± 4.6 | **<0.001** | 12.1 ± 3.6 | 8.5 ± 4.7 | **<0.001** |
| Age group on index date (years), n (%) | | | | | | |
| ≤11 | 66 (53.7) | 274 (68.3) | **0.002** | 29 (44.6) | 311 (67.8) | **0.001** |
| 12–17 | 57 (46.3) | 127 (31.7) |  | 36 (55.4) | 148 (32.2) |  |
| Gender | | | | | | |
| Female | 67 (54.5) | 240 (59.9) | 0.514 | 38 (58.5) | 269 (58.6) | 0.628 |
| Male | 55 (44.7) | 159 (39.7) |  | 26 (40.0) | 188 (41.0) |  |
| Unknown | <3 | <3 |  | <3 | <3 |  |
| Year started burosumab (Index year), n (%) | | | | | | |
| 2018 | 11 (8.9) | 43 (10.7) | **0.043** | 10 (15.4) | 44 (9.6) | 0.269 |
| 2019 | 53 (43.1) | 123 (30.7) |  | 25 (38.5) | 151 (32.9) |  |
| 2020 | 15 (12.2) | 62 (15.5) |  | 7 (10.8) | 70 (15.3) |  |
| 2021 | 15 (12.2) | 73 (18.2) |  | 10 (15.4) | 78 (17.0) |  |
| 2022 | 14 (11.4) | 64 (16.0) |  | 5 (7.7) | 73 (15.9) |  |
| 2023 | 15 (12.2) | 30 (7.5) |  | 8 (12.3) | 37 (8.1) |  |
| 2024 | 0 (0.0) | 6 (1.5) |  | 0 (0.0) | 6 (1.3) |  |
| Geographic region, n (%) | | | | | | |
| Midwest | 35 (28.5) | 99 (24.7) | 0.140 | 23 (35.4) | 111 (24.2) | 0.133 |
| Northeast | 15 (12.2) | 86 (21.4) |  | 7 (10.8) | 94 (20.5) |  |
| South | 53 (43.1) | 163 (40.6) |  | 27 (41.5) | 189 (41.2) |  |
| West | 20 (16.3) | 53 (13.2) |  | 8 (12.3) | 65 (14.2) |  |
| Unknown | 0 (0.0) | 0 (0.0) |  | 23 (35.4) | 111 (24.2) |  |
| Payer channel, n (%) | | | | | | |
| Commercial | 31 (25.2) | 155 (38.7) | 0.050 | 24 (36.9) | 162 (35.3) | 0.312 |
| Medicaid | 90 (73.2) | 242 (60.3) |  | 39 (60.0) | 293 (63.8) |  |
| Medicare Advantage/FFS | <3 | <3 |  | <3 | <3 |  |
| Other/unknown | <3 | 3 (0.7) |  | <3 | 3 (0.7) |  |
| Race, n (%) | | | | | | |
| Asian or Pacific Islander | 4 (3.3) | 8 (2.0) | **<0.001** | <3 | 10 (2.2) | 0.176 |
| Black or African American | 20 (16.3) | 52 (13.0) |  | 11 (16.9) | 61 (13.3) |  |
| Hispanic or Latino | 34 (27.6) | 48 (12.0) |  | 11 (16.9) | 71 (15.5) |  |
| White | 52 (42.3) | 177 (44.1) |  | 33 (50.8) | 196 (42.7) |  |
| Other/unknown | 13 (10.6) | 116 (28.9) |  | 8 (12.3) | 121 (26.4) |  |
| ^a^Patients with claims for opioids could also have claims for non-opioids.  Wilcoxon rank sum test; Pearson’s Chi-squared test with simulated P value (based on 2000 replicates); statistically significant findings indicated in **bold**.  Payer channel categories were mutually exclusive.  Counts <3 were masked to comply with HIPAA regulations.  FFS, fee for service; HIPAA, Health Insurance Portability and Accountability Act; PPM, prescription pain medication; SD, standard deviation; XLH, X-linked hypophosphatemia. | | | | | | |

Supplementary Table 4. Bivariate analysis: baseline (pre-treatment) clinical characteristics of pediatric (<18 years) patients with XLH who received burosumab treatment according to PPM claims during baseline (PPM vs no PPM; opioid vs no opioid)

|  | | | PPM  (n=123) | No PPM  (n=401) | P value | Opioids  (n=65) | No opioids  (n=459) | p value |
| --- | --- | --- | --- | --- | --- | --- | --- | --- |
| **CCI score,** **mean ± SD** | | | 0.4 ± 0.8 | 0.1 ± 0.6 | **<0.001** | 0.3 ± 0.7 | 0.2 ± 0.7 | **0.020** |
| CCI comorbidities, n (%)^b^ | | | | | | | | |
| Any malignancy | | | <3 | <3 | 0.132 | <3 | <3 | **0.040** |
| Chronic pulmonary disease | | | 26 (21.1) | 22 (5.5) | **<0.001** | 10 (15.4) | 38 (8.3) | 0.077 |
| Renal disease | | | 7 (5.7) | 8 (2.0) | **0.048** | 3 (4.6) | 12 (2.6) | 0.428 |
| Musculoskeletal manifestations, n (%) | | | | | | | | |
| Osteoarthritis | | | 4 (3.3) | 6 (1.5) | 0.266 | 3 (4.6) | 7 (1.5) | 0.132 |
| Arthralgia | | | 73 (59.3) | 129 (32.2) | **<0.001** | 45 (69.2) | 157 (34.2) | **<0.001** |
| Fracture | | | 32 (26.0) | 30 (7.5) | **<0.001** | 19 (29.2) | 43 (9.4) | **<0.001** |
| Myalgia | | | 7 (5.7) | 18 (4.5) | 0.616 | 3 (4.6) | 22 (4.8) | >0.999 |
| Spinal stenosis | | | <3 | 3 (0.7) | 0.617 | <3 | 4 (0.9) | >0.999 |
| Enthesopathy | | | 10 (8.1) | 8 (2.0) | **0.004** | 9 (13.8) | 9 (2.0) | **<0.001** |
| Kyphosis | | | <3 | <3 | >0.999 | <3 | <3 | 0.299 |
| Scoliosis | | | 16 (13.0) | 41 (10.2) | 0.416 | 9 (13.8) | 48 (10.5) | 0.496 |
| Non-musculoskeletal manifestations, n (%) | | | | | | | | |
| Hypertension | | | 12 (9.8) | 25 (6.2) | 0.227 | 5 (7.7) | 32 (7.0) | >0.999 |
| Obesity | | | 51 (41.5) | 121 (30.2) | **0.022** | 26 (40.0) | 146 (31.8) | 0.207 |
| Depression | | | 31 (25.2) | 41 (10.2) | **<0.001** | 19 (29.2) | 53 (11.5) | **<0.001** |
| Deformity-related conditions, n (%) | | | | | | | | |
| Chiari malformation | | | 6 (4.9) | 9 (2.2) | 0.207 | 4 (6.2) | 11 (2.4) | 0.109 |
| Craniosynostosis | | | 11 (8.9) | 32 (8.0) | 0.862 | 6 (9.2) | 37 (8.1) | 0.805 |
| Delayed growth/delayed walking | | | 24 (19.5) | 75 (18.7) | 0.893 | 12 (18.5) | 87 (19.0) | >0.999 |
| Short stature | | | 58 (47.2) | 165 (41.1) | 0.252 | 29 (44.6) | 194 (42.3) | 0.797 |
| Hip/leg-related deformities^c^ | | | 103 (83.7) | 270 (67.3) | **<0.001** | 62 (95.4) | 311 (67.8) | **<0.001** |
| Rickets | | | 116 (94.3) | 318 (79.3) | **<0.001** | 64 (98.5) | 370 (80.6) | **0.001** |
| Other symptoms and conditions of interest, n (%) | | | | | | | | |
| Vitamin D deficiency | | | 45 (36.6) | 134 (33.4) | 0.608 | 21 (32.3) | 158 (34.4) | 0.779 |
| Dental complications | | | 45 (36.6) | 123 (30.7) | 0.234 | 24 (36.9) | 144 (31.4) | 0.387 |
| Difficulty walking | | | 41 (33.3) | 93 (23.2) | **0.028** | 25 (38.5) | 109 (23.7) | **0.015** |
| Muscle weakness | | | 15 (12.2) | 46 (11.5) | 0.869 | 11 (16.9) | 50 (10.9) | 0.215 |
| Hearing loss | | | 23 (18.7) | 52 (13.0) | 0.137 | 11 (16.9) | 64 (13.9) | 0.566 |
| Hyperparathyroidism | | | 4 (3.3) | 21 (5.2) | 0.447 | <3 | 23 (5.0) | 0.554 |
| Kidney stone | | | 7 (5.7) | 12 (3.0) | 0.175 | <3 | 17 (3.7) | >0.999 |
| Nephrocalcinosis | | | 20 (16.3) | 58 (14.5) | 0.667 | 13 (20.0) | 65 (14.2) | 0.270 |
| Osteomalacia | | | 0 (0.0) | 3 (0.7) | 0.587 | 0 (0.0) | 3 (0.7) | >0.999 |
| Tinnitus | | | <3 | 3 (0.7) | 0.607 | <3 | 4 (0.9) | >0.999 |
| Vertigo | | | 0 (0.0) | <3 | >0.999 | 0 (0.0) | <3 | >0.999 |
| Treatments, n (%) | | | | | | | | |
| Phosphate salts/active vitamin D | Calcitriol | | 102 (82.9) | 268 (66.8) | **<0.001** | 55 (84.6) | 315 (68.6) | **0.006** |
|  | Phosphate supplements | | 62 (50.4) | 155 (38.7) | **0.023** | 34 (52.3) | 183 (39.9) | 0.065 |
| Other conventional vitamin D | Cholecalciferol | | 46 (37.4) | 79 (19.7) | **<0.001** | 20 (30.8) | 105 (22.9) | 0.207 |
| Pain-related healthcare service utilization | | | | | | | | |
| Physical therapy | | Patients with visits, n (%) | 46 (37.4) | 49 (12.2) | **<0.001** | 35 (53.8) | 60 (13.1) | **<0.001** |
|  |  | Number of visits, mean ± SD | 13.7 ± 15.0 | 10.8 ± 14.4 | 0.516 | 11.5 ± 12.3 | 12.6 ± 16.0 | 0.745 |
| Occupational therapy | | Patients with visits, n (%) | 7 (5.7) | 13 (3.2) | 0.285 | 3 (4.6) | 17 (3.7) | 0.722 |
|  |  | Number of visits, mean ± SD | 1.3 ± 0.5 | 1.5 ± 1.4 | 0.917 | 1.0 ± 0.0 | 1.5 ± 1.2 | 0.327 |
| Alternative medicine^d^ | | Patients with visits, n (%) | <3 | 4 (1.0) | 0.636 | <3 | 5 (1.1) | >0.999 |
|  |  | Number of visits, mean ± SD | 5.0 ± 4.2 | 7.5 ± 4.8 | 0.573 | 8.0 ± NA | 6.4 ± 4.8 | NA |
| ^a^Patients with claims for opioids could also have claims for non-opioids.  ^b^CCI comorbidities with claims by ≥5% of patients in either age group, or that were significant, are reported.  ^c^Genu varum, genu valgum, varus deformities, and coxa vara.  ^d^Acupuncture and chiropractic services.  Clinical characteristics were assessed during baseline (the 12 months before the first prescription for burosumab [the index date]) for patients with continuous medical and pharmacy benefits during that period.  Wilcoxon rank sum test; Pearson’s Chi-squared test with simulated P value (based on 2000 replicates); statistically significant findings indicated in **bold**.  Counts <3 were masked to comply with HIPAA regulations.  CCI, Charlson Comorbidity Index; HIPAA, Health Insurance Portability and Accountability Act; NA, not applicable; PPM, prescription pain medication; SD, standard deviation; XLH, X-linked hypophosphatemia. | | | | | | | | |

Supplementary Table 5. Baseline (pre-treatment) demographic characteristics for adults with XLH who received burosumab treatment and who had 12 months’ and 24 months’ follow-up data

|  | | Patients with 12 months’ follow-up  (n=288) | Patients with 24 months’ follow-up  (n=214) |
| --- | --- | --- | --- |
| **Age, years** | Mean ± SD | 37.6 ± 14.7 | 38.0 ± 15 |
|  | Range | 18–80 | 18–80 |
| Age group, years, n (%) | | | |
| 18–29 | | 104 (36.1) | 76 (35.5) |
| 30–39 | | 55 (19.1) | 37 (17.3) |
| 40–49 | | 66 (22.9) | 53 (24.8) |
| ≥50 | | 63 (21.9) | 48 (22.4) |
| Sex, n (%) | | | |
| Female | | 191 (66.3) | 144 (67.3) |
| Male | | 91 (31.6) | 66 (30.8) |
| Unknown | | 6 (2.1) | 4 (1.9) |
| Year started burosumab (index year), n (%) | | | |
| 2018^a^ | | 27 (9.4) | 26 (12.2) |
| 2019 | | 103 (35.8) | 91 (42.5) |
| 2020 | | 64 (22.2) | 52 (24.3) |
| 2021 | | 52 (18.1) | 40 (18.7) |
| 2022 | | 38 (13.2) | 5 (2.3) |
| 2023 | | 4 (1.4) | 0 (0.0) |
| Region, n (%) | | | |
| Northeast | | 67 (23.3) | 49 (22.9) |
| Midwest | | 73 (25.3) | 59 (27.6) |
| South | | 101 (35.1) | 73 (34.1) |
| West | | 46 (16.0) | 32 (15.0) |
| Unknown | | ≤3 | ≤3 |
| Payer channel, n (%)^b^ | | | |
| Commercial | | 182 (63.2) | 138 (64.5) |
| Managed Medicaid/Medicaid | | 76 (26.4) | 49 (22.9) |
| Medicare Advantage/FFS | | 28 (9.7) | 25 (11.7) |
| Other/unknown | | ≤3 | ≤3 |
| Race and ethnicity, n (%) | | | |
| Asian or Pacific Islander | | 6 (2.1) | 6 (2.8) |
| Black or African American | | 24 (8.3) | 14 (6.5) |
| Hispanic or Latino | | 35 (12.2) | 26 (12.2) |
| White | | 154 (53.5) | 116 (54.2) |
| Other/unknown | | 69 (24.0) | 52 (24.3) |
| ^a^2018 was an incomplete year.  ^b^Payer channel categories were mutually exclusive.  Counts ≤3 have been masked to comply with HIPAA regulations.  FFS, fee for service; HIPAA, Health Insurance Portability and Accountability Act; SD, standard deviation; XLH, X-linked hypophosphatemia. | | | |

Supplementary Table 6. Baseline (pre-treatment) clinical characteristics for adults with XLH who received burosumab treatment and who had 12 months’ and 24 months’ follow-up data

|  | Patients with 12 months’ follow-up  (n=288) | Patients with 24 months’ follow-up  (n=214) |
| --- | --- | --- |
| CCI score | | |
| Mean ± SD | 0.4 ± 1.0 | 0.4 ± 0.9 |
| Range | 0–8 | 0–5 |
| CCI comorbidities, n (%)^a^ | | |
| Chronic pulmonary disease | 40 (13.9) | 30 (14.0) |
| Diabetes without complications | 16 (5.6) | 12 (5.6) |
| Renal disease | 18 (6.3) | 10 (4.7) |
| Musculoskeletal manifestations, n (%) | | |
| Arthralgia | 109 (37.8) | 76 (35.5) |
| Enthesopathy | 31 (10.8) | 26 (12.2) |
| Fracture | 45 (15.6) | 33 (15.4) |
| Kyphosis | ≤3 | ≤3 |
| Myalgia | 16 (5.6) | 13 (6.1) |
| Osteoarthritis | 91 (31.6) | 68 (31.8) |
| Scoliosis | 12 (4.2) | 10 (4.7) |
| Spinal stenosis | 21 (7.3) | 16 (7.5) |
| Non-musculoskeletal manifestations, n (%) | | |
| Depression | 72 (25.0) | 54 (25.2) |
| Hypertension | 82 (28.5) | 58 (27.1) |
| Obesity | 105 (36.5) | 78 (36.4) |
| Deformity-related conditions, n (%) | | |
| Chiari malformation | 6 (2.1) | 4 (1.9) |
| Craniosynostosis | 0 (0.0) | 0 (0.0) |
| Delayed growth/delayed walking | ≤3 | ≤3 |
| Hip/leg-related deformities^b^ | 24 (8.3) | 19 (8.9) |
| Rickets^c^ | 120 (41.7) | 92 (43.0) |
| Short stature | 15 (5.2) | 11 (5.1) |
| Other symptoms and conditions of interest, n (%) | | |
| Dental complications | 21 (7.3) | 12 (5.6) |
| Difficulty walking | 26 (9.0) | 21 (9.8) |
| Hearing loss | 25 (8.7) | 19 (8.9) |
| Hyperparathyroidism | 33 (11.5) | 24 (11.2) |
| Kidney stone | 9 (3.1) | 6 (2.8) |
| Muscle weakness | 16 (5.6) | 11 (5.1) |
| Nephrocalcinosis | 11 (3.8) | 8 (3.7) |
| Osteomalacia | 25 (8.7) | 19 (8.9) |
| Tinnitus | 14 (4.9) | 9 (4.2) |
| Vertigo | 4 (1.4) | 4 (1.9) |
| Vitamin D deficiency | 81 (28.1) | 56 (26.2) |
| Treatments, n (%) | | |
| Calcitriol | 138 (47.9) | 111 (51.9) |
| Phosphate supplements | 83 (28.8) | 60 (28.0) |
| Cholecalciferol | 17 (5.9) | 11 (5.1) |
| ^a^CCI comorbidities with claims by ≥5% in Table 2 are reported.  ^b^Genu varum, genu valgum, varus deformities, and coxa vara.  ^c^Several diagnostic codes relating to disorders of bone mineralization are not age-restricted and may appear in adult claims data, such as E83.31 (Vitamin D-resistant rickets), E55.0 (active rickets [nutritional/vitamin D deficiency]) and E64.3 (sequelae [inactive] rickets]). These may represent sequelae from prior disease, persistence of childhood disease manifestations, late diagnosis or miscoding.  Clinical characteristics were assessed during baseline (the 12 months before the first prescription for burosumab [the index date]) for patients with continuous medical and pharmacy benefits during that period.  Counts ≤3 have been masked to comply with HIPAA regulations.  CCI, Charlson Comorbidity Index; HIPAA, Health Insurance Portability and Accountability Act; SD, standard deviation; XLH, X-linked hypophosphatemia. | | |

Supplementary Table 7. Changes in pain medication use: number of days covered by claims for opioid prescription and dose

|  | Patients with 12 months’ follow-up | | Patients with 24 months’ follow-up | |
| --- | --- | --- | --- | --- |
|  | Baseline | Follow-up | Baseline | Follow-up |
| Adults | | | | |
| n | 288 | | 214 | |
| Number of days’ opioid prescription^a^ | 99 ± 135.3 | 133 ± 146.8 | 109 ± 141.5 | 120 ± 149.8 |
| Dose^b^ | 1,235 ± 2448.0 | 869 ± 917.9 | 1,197 ± 2404.2 | 986 ± 1014.4 |
| Pediatric patients | | | | |
| n | 412 | | 322 | |
| Number of days’ opioid prescription^a^ | 8 ± 8.9 | 7 ± 8.6 | 9 ± 9.1 | 5 ± 6.1 |
| Dose^b^ | 866 ± 1318.5 | 573 ± 955.7 | 780 ± 1276 | 785 ± 1042.9 |
| ^a^Total number of unique days on which a patient had an active prescription for opioid pain medication, excluding any overlapping days between prescriptions, mean ± SD.  ^b^Morphine equivalent daily dose (mg), mean ± SD.  SD, standard deviation. | | | | |

Supplementary Table 8. Baseline (pre-treatment) demographic characteristics for pediatric patients with XLH who received burosumab treatment and who had 12 months’ and 24 months’ follow-up data

|  | | Patients with 12 months’  follow-up  (n=412) | Patients with 24 months’  follow-up  (n=322) |
| --- | --- | --- | --- |
| **Age, years** | Mean ± SD | 9.1 ± 4.7 | 9.3 ± 4.6 |
|  | Range | 1–17 | 1–17 |
| Age group, years, n (%) | | | |
| ≤11 | | 258 (62.6) | 195 (60.6) |
| 12–17 | | 154 (37.4) | 127 (39.4) |
| **Sex, n (%)** | | | |
| Female | | 244 (59.2) | 191 (59.3) |
| Male | | 166 (40.3) | 129 (40.1) |
| Unknown | | ≤3 | ≤3 |
| Year started burosumab (index year), n (%) | | | |
| 2018^a^ | | 53 (12.9) | 48 (14.9) |
| 2019 | | 162 (39.3) | 156 (48.4) |
| 2020 | | 74 (18.0) | 59 (18.3) |
| 2021 | | 81 (19.7) | 55 (17.1) |
| 2022 | | 37 (9.0) | 4 (1.2) |
| 2023 | | 5 (1.2) | 0 (0.0) |
| Region, n (%) | | | |
| Northeast | | 81 (19.7) | 64 (19.9) |
| Midwest | | 100 (24.3) | 81 (25.2) |
| South | | 177 (43.0) | 139 (43.2) |
| West | | 54 (13.1) | 38 (11.8) |
| Unknown | | 0 (0.0) | 0 (0.0) |
| Payer channel, n (%)^b^ | | | |
| Commercial | | 147 (35.7) | 120 (37.3) |
| Managed Medicaid/Medicaid | | 263 (63.8) | 200 (62.1) |
| Medicare Advantage/FFS | | ≤3 | ≤3 |
| Other/unknown | | 0 (0.0) | 0 (0.0) |
| Race and ethnicity, n (%) | | | |
| Asian or Pacific Islander | | 9 (2.2) | 6 (1.9) |
| Black or African American | | 60 (14.6) | 46 (14.3) |
| Hispanic or Latino | | 63 (15.3) | 48 (14.9) |
| White | | 185 (44.9) | 153 (47.5) |
| Other/unknown | | 95 (23.1) | 69 (21.4) |
| ^a^2018 was an incomplete year.  ^b^Payer channel categories were mutually exclusive.  Counts ≤3 have been masked to comply with HIPAA regulations.  FFS, fee for service; HIPAA, Health Insurance Portability and Accountability Act; SD, standard deviation; XLH, X-linked hypophosphatemia. | | | |

Supplementary Table 9. Baseline (pre-treatment) clinical characteristics for pediatric patients with XLH who received burosumab treatment and who had 12 months’ and 24 months’ follow-up data

|  | Patients with 12 months’  follow-up  (n=412) | Patients with 24 months’  follow-up  (n=322) |
| --- | --- | --- |
| CCI score | | |
| Mean ± SD | 0.2 ± 0.8 | 0.2 ± 0.8 |
| Range | 0–11 | 0–11 |
| CCI comorbidities, n (%)^a^ | | |
| Chronic pulmonary disease | 38 (9.2) | 35 (10.9) |
| Diabetes without complications | 4 (1.0) | 4 (1.2) |
| Renal disease | 14 (3.4) | 12 (3.7) |
| Musculoskeletal manifestations, n (%) | | |
| Arthralgia | 57 (13.8) | 52 (16.1) |
| Enthesopathy | ≤3 | ≤3 |
| Fracture | 13 (3.2) | 13 (4.0) |
| Kyphosis | 0 (0.0) | 0 (0.0) |
| Myalgia | 4 (1.0) | 4 (1.2) |
| Osteoarthritis | ≤3 | ≤3 |
| Scoliosis | 17 (4.1) | 16 (5.0) |
| Spinal stenosis | ≤3 | ≤3 |
| Non-musculoskeletal manifestations, n (%) | | |
| Depression | 20 (4.9) | 16 (5.0) |
| Hypertension | 7 (1.7) | 4 (1.2) |
| Obesity | 69 (16.7) | 57 (17.7) |
| Deformity-related conditions, n (%) | | |
| Chiari malformation | ≤3 | ≤3 |
| Craniosynostosis | 18 (4.4) | 17 (5.3) |
| Delayed growth/delayed walking | 36 (8.7) | 32 (9.9) |
| Hip/leg-related deformities^b^ | 209 (50.7) | 168 (52.2) |
| Rickets | 228 (55.3) | 188 (58.4) |
| Short stature | 98 (23.8) | 85 (26.4) |
| Other symptoms and conditions of interest, n (%) | | |
| Dental complications | 41 (10.0) | 31 (9.6) |
| Difficulty walking | 54 (13.1) | 44 (13.7) |
| Hearing loss | 19 (4.6) | 18 (5.6) |
| Hyperparathyroidism | 7 (1.7) | 6 (1.9) |
| Kidney stone | ≤3 | ≤3 |
| Muscle weakness | 18 (4.4) | 18 (5.6) |
| Nephrocalcinosis | 33 (8.0) | 30 (9.3) |
| Osteomalacia | 0 (0.0) | 0 (0.0) |
| Tinnitus | 0 (0.0) | 0 (0.0) |
| Vertigo | 0 (0.0) | 0 (0.0) |
| Vitamin D deficiency | 39 (9.5) | 25 (7.8) |
| Treatments, n (%) | | |
| Calcitriol | 254 (61.7) | 212 (65.8) |
| Phosphate supplements | 130 (31.6) | 106 (32.9) |
| Cholecalciferol | 41 (10.0) | 28 (8.7) |
| ^a^CCI comorbidities with claims by ≥5% in Table 2 are reported.  ^b^Genu varum, genu valgum, varus deformities, and coxa vara.  Clinical characteristics were assessed during baseline (the 12 months before the first prescription for burosumab [the index date]) for patients with continuous medical and pharmacy benefits during that period.  Counts ≤3 have been masked to comply with HIPAA regulations.  CCI, Charlson Comorbidity Index; HIPAA, Health Insurance Portability and Accountability Act; SD, standard deviation; XLH, X-linked hypophosphatemia. | | |
